# Ophthalmology

**Published:** 1894-01-06

**Authors:** Clements Hailes

**Affiliations:** Hon. Assistant Surgeon to the Bristol Eye Hospital


### OPHTHALMOLOGY.

### On Blepharitis, its Causes and Treatment.

By Clements Hailes, M.D.Edin., Hon. Assistant Surgeon to the Bristol Eye Hospital.

When a patient presents himself complaining of "weak eyes" or of being "tender-eyed" he generally means to indicate that he is suffering from chronic conjunctivitis, or that the borders of the eyelids, the ciliary margins, are inflamed, or that the two conditions co-exist. That is the term commonly used in these parts in contra-distinction to "weak eyesight." This condition is called Blepharitis, Tinea Tarsi, or sometime Ophthalmia Tarsi.

Generally both eyelids of both eyes participate in the affection, but the complaint may be confined to one, and may only amount to a redness at the edges of the eyelids, or the margins may be covered with thick crusts or scabs protecting small ulcers; or again, the eye-lashes may be destroyed, and the thickened and everted conjunctiva present a raw appearance. Whichever condition exist it is most unsightly. Blepharitis commonly occurs in children, but is often seen in adults. Its first appearance may date from some illness, such as, and most commonly, measles, whooping cough, or scarlet fever, the patients being left by these diseases in an unhealthy anæmic condition and state of inanition. Unwholesome or poor living, with unsuitable, or too little food, and the strumous diathesis also predispose to it.

Irritating discharges from the conjunctiva are also a fertile cause of blepharitis, and so it accompanies diseases of the conjunctiva, or other forms of eye disease, from errors of refraction, overuse of the eyes, exposure to wind, dirt, and also by pediculi finding a habitat amongst the cilia. It consists of a chronic inflammation of the ciliary margin of the eyelids and the numerous glands connected with it where the mucous membrance joins the skin at the margins of the eyelids, and of the hair follicles and sebaceous glands at the skin margin, the secretions become altered in character, decompose from retention, eventually escaping acrid and irritating, dry up into scabs, and block up the exits of other glands, causing their products in turn to be retained and decompose, and add their irritating properties. The thickening of the mucous membrane may displace the punctum lachrymalis, so that the tears flow over and assist in the general irritant effect. The eyelashes become stunted and fall out, little pustules form along the edges of the eyelids, and bursting add to the other secretions.

When in an adult there is merely redness of the edges, it is probably due to stasis in the blood vessels at the margins of the lids brought about by unwonted efforts of accommodation in an ametropic eye, and one's attention should be directed to ascertaining the refraction of the eye and the occupation of the patient, for it often occurs in persons with hypermetropia or astigmatism, even in low degrees, who are occupied with work of a minute nature, calling for efforts at accommodation for near vision. Thus it is often found amongst children who have just begun to go to school or to read and write, in those who work with the needle, boot finishers, folders, clerks, students reading, artists, engine fitters, &c. In all this class of case where hypermetropia or astigmatism is found, the prescribing and wearing of suitable spectacles at work will soon cause the redness to disappear. If, however, no error of refraction exist, which is occasionally the case, the constitutional causes must be sought for; sometimes a sexual irregularity will account for it. Attention to diet, change of air, tonics, more especially ferruginous ones, continued for some length of time, are beneficial.

When the eyelid margins are covered with scabs, applications to soften the latter and counteract the irritating secretions, and stimulate the glands, are called for, and hence it is that a quack preparation called Singleton's Golden Eye Ointment has such a great reputation among the British public. It is supposed to contain the red oxide of mercury crystals powdered down, and is no better than the yellow ointment of Dr. Pagenstecher, which is less likely to irritate, as it contains the yellow oxide of mercury. In ophthalmic practice various modifications of it are used. These consist of the yellow oxide of mercury in vaselin, gr. ss., gr. j., grs. ij., or grs. iij. of the oxide to each ʒj. of vaselin, and in cases where an error of refraction exists it is advantageously made up with some sulphate of atropia alkaloid added to it—from one-fourth of a gr. to gr. j. in the ʒj. of vaselin is enough. It is best, however, not to trust to this for removing the scabs, but to order an alkaline bath for the eyelids; ʒj. of sodii bicarb. in a pint of warm water does well, or the sodii biborat in the same proportions dissolved in water, and, having removed the scabs, then apply the ointment. It may be necessary to remove the eyelashes, and continue treatment for some time, but often the result is attained rapidly.

Where the conjunctiva is thickened and everted, and exuding an irritating secretion which excoriates the skin below, producing "eczema tarsi," I get excellent results from painting the conjunctiva every second or third day with a strong solution of perchloride of mercury, 1 in 250, and letting the patient use a weaker one, 1 in 5,000 generally, every few hours, using no ointment or greasy substance, so that the solution shall at each application find its way into the folds and follicles of the diseased mucous membrane. Lapis divinus, or bluestone, solid or in solution, are useful in these cases. Mercurial ointments, after or without epilation, are the most efficacious remedies for removing the pediculi when present. Blepharitis must not be considered a trifling complaint for it is often a warning of other complaints and is always a source of distress on account of its disfigurement. Patients are generally willing to persevere with treatment, and results are good, as except in very severe cases the condition may be much alleviated and the eyelids regain their normal appearance with even the cilia restored in the milder cases. It is very important to remember that besides the local and constitutional treatment, attention must be paid to the refraction, and if errors exist suitable glasses must be used, or there will be a great tendency to relapses. The refraction is best ascertained by the aid of retinoscopy, after the pupils have been dilated and the accommodation paralysed by the use of atropine drops or ointment. There has lately been an outcry about the use of atropine as it prevents these patients from following their employment for some days, and it has been advised that homatropine should be used instead, to discover the latent hypermetropia. Personally I have found homatropine unsatisfactory in several cases, owing to a want of thorough paralysis of accommodation; the retinoscopy works out as one of astigmatism, and yet no cylinder can be borne some days afterwards. This, I believe, can be avoided if the drops are sufficiently strong, and are conscientiously used every two or three hours for twenty-four hours. I find, however, that in those cases that cannot afford the loss of time, and whose vision can be brought up to the normal standard of vision with plus glasses, *e.g.*, when the manifest hypermetropia can be corrected, it is best to prescribe the glass that corrects it, leaving out of account the latent hypermetropia, but where there is any ciliary spasm, and the hypermetropia cannot be corrected as above, then atropine is the only agent that will give complete satisfaction, and its use will well repay the patient for his loss of time. It of course seems cruel to keep the patient from earning his livelihood, but in these cases he is certain in time to reach the point at which he cannot go on, and in the meanwhile to suffer from headaches and dimness of vision, whereas a few days of atropine "blindness," followed by the prescription of suitable glasses, and he returns to work with renewed vigour.

